# Before diagnosing SARS-CoV-2-related juvenile ischemic stroke, a causal link needs to be established

**DOI:** 10.11604/pamj.2023.46.83.36740

**Published:** 2023-11-13

**Authors:** Josef Finsterer

**Affiliations:** 1 Neurology and Neurophysiology Center, Vienna, Austria

**Keywords:** COVID-19, SARS-CoV-2, juvenile stroke, encephalitis, meningitis

## To the editors of the Pan African Medical Journal

We read with interest the article by Osman *et al*. about a 6.5 years old male who was admitted for fever, abdominal pain, vomiting, diarrhoea, conjunctivitis, generalised rashes, reduced urinary frequency, and poor appetite [1]. Consecutively, he developed a headache, irritability, loss of speech, and right facial weakness [1]. A neurological exam was noteworthy for drowsiness, neck stiffness, aphasia, and right hemiparesis [1]. Since the patient had been exposed to SARS-CoV-2 one week prior to admission, SARS-CoV-2-related meningitis was suspected but not confirmed by cerebrospinal fluid (CSF) studies [1]. Surprisingly, a cerebral MRI revealed an ischemic stroke in the area of the left middle cerebral artery territory [1]. The study is appealing but raises concerns that should be discussed.

We disagree with the notion that the patient had SARS-CoV-2. The nasopharyngeal swab test was negative and there was no detection of viral RNA in CSF [1]. Serum neutralising IgG antibodies were positive, but exposure to a SARS-CoV-2 infected relative was only one week prior to admission. Since neutralising IgG antibodies are detectable in serum not earlier than 14-21 days after exposure to the virus [2], it is rather unlikely that the patient contracted the virus from his infected relative. A third argument against SARS-CoV-2 infection as the cause of ischemic stroke is that the patient did not develop respiratory symptoms.

Another limitation of the study is that the patient was diagnosed with meningitis/encephalitis, although the CSF neither showed pleocytosis nor carried nucleic acid from any infectious agent. The CSF was obviously not tested for SARS-CoV-2. We should be told if cerebral MRI showed an area of enhancement after application of gadolinium. A further argument against meningitis/encephalitis is that the Brudzinski sign was negative and that the patient did not complain about oversensitivity to light or noise.

A further limitation is that the patient underwent lumbar puncture before cerebral imaging. Because it is mandatory to rule out a structural lesion as cause of a central nervous system manifestation before considering meningitis/encephalitis, it is recommended that imaging precedes lumbar puncture in all cases. No explanation was provided for elevated erythrocytes in the CSF. Was this due to traumatic lumbar puncture or did the erythrocytes originate from bleeding due to traumatic lumbar puncture? In this respect, we should be informed if the CSF was positive or negative for bilirubin and hemosiderin. Since the CSF was positive for erythrocytes, subarachnoid bleeding, and intra-cerebral bleeding need to be carefully ruled out. Although multimodal MRI showed cytotoxic edema, the patient did not necessarily need to have an ischemic stroke. Differentials that should be considered and ruled out before diagnosing ischemic stroke include acute, hemorrhagic leucoencephalitis (AHLE), acute, hemorrhagic, necrotising encephalitis (AHNE), posterior reversible encephalopathy syndrome (PRES), acute, disseminated encephalo-myelitis (ADEM), cerebral vasculitis, reversible, cerebral vasoconstriction syndrome (RCVS), and venous sinus thrombosis (VST).

Assuming that the child truly experienced an ischemic stroke, we should be informed about the pathophysiology of stroke. Was it due to macro-angiopathy, micro-angiopathy, due to cardio-embolism, coagulopathy, or due to disturbed thrombocyte functions? Assuming embolic pathogenesis, we should be informed if atrial or ventricular thrombus formation, ventricular arrhythmias, atrial fibrillation, heart failure, non-compaction, and Takotsubo syndrome (TTS) have been appropriately ruled out. Were drugs such as ibuprofen considered as causes of ischemic stroke? From non-steroidal anti-inflammatory drugs (NSAIDs) it is known that they can be pro-thrombotic [3]. NSAIDs cause a pro-thrombotic state by inhibiting coagulation through inhibiting the synthesis of thromboxane and of prostacyclin [3]. NSAID also reduces the production of prostacyclines resulting in vasoconstriction of arterioles with prolonged reduction in afferent blood flow [4].

There is a discrepancy between the clinical abdominal exam described as “unremarkable” and the symptoms on admission, such as abdominal pain, vomiting, poor appetite, and diarrhoea [1]. This discrepancy should be solved. Did the abdominal symptoms disappear prior to the clinical exam? Overall, the interesting study has limitations that call the results and their interpretation into question. Clarifying these weaknesses would strengthen the conclusions and could improve the study. Before diagnosing SARS-CoV-2-related ischemic stroke, various differentials need to be ruled out and if SARS-CoV-2-related stroke is confirmed, the pathophysiology of SARS-CoV-2-related juvenile stroke should be elucidated to prevent a relapse.

## References

[1] Osman RS, Dawood SS, Thawer SP, Mandania SM, Amirali MH, Bulimba MN (2022). SARS-CoV-2 precipitating a stroke in a child? A case report from Tanzania. Pan Afr Med J.

[2] Universität Zürich (2022). Institut für Medizinische Virologie. Diagnostik, Antikörpernachweis.

[3] Auriel E, Regev K, Korczyn AD (2014). Nonsteroidal anti-inflammatory drugs exposure and the central nervous system. Handb Clin Neurol.

[4] Harris RC (2006). COX-2 and the kidney. J Cardiovasc Pharmacol.

